# Meal frequency strategies for the management of type 2 diabetes subjects: A systematic review

**DOI:** 10.1371/journal.pone.0298531

**Published:** 2024-02-29

**Authors:** Roxana Paola Gómez-Ruiz, Abraham Isaí Cabello-Hernández, Francisco Javier Gómez-Pérez, Miguel Ángel Gómez-Sámano

**Affiliations:** Department of Endocrinology and Lipid Metabolism, Instituto Nacional de Ciencias Medicas y Nutrición Salvador Zubirán, Mexico City, Mexico; Dynamical Business & Science Society - DBSS International SAS, COLOMBIA

## Abstract

**Background:**

Effective nutrition management is fundamental in the comprehensive treatment of individuals with type 2 diabetes. Various strategies have been explored in this regard, demonstrating their potential usefulness in improving clinical outcomes. This systematic review aims to assess the impact of meals frequency on the well-being of these patients.

**Methods and findings:**

In accordance with PRISMA (Preferred Reporting Items for Systematic Reviews and Meta–Analyses) guidelines, PubMed, Embase, Web of Science, Cochrane Library, and Google Scholar databases were searched until July 10th, 2023. We included studies from the last 10 years in people with type 2 diabetes that had an intervention regarding their meal frequency. The risk of bias was evaluated based on the Cochrane tool according to the type of study. Of 77 retrieval articles, 13 studies matched our inclusion criteria. The primary focus of each study was to evaluate glycemic control as the major outcome. Studies suggest that meal frequency, time-restricted feeding, breakfast skipping, bedtime snacking, and chrononutrition practices all play roles in type 2 diabetes management and risk.

**Conclusions:**

Restricting feeding to 2 to 3 meals per day and practicing time restricted feeding with less than 10 hours of daily food intake promotes weight loss and glycemic control in patients with type 2 diabetes. Aligning food consumption with the body’s natural rhythm is beneficial, whereas skipping breakfast disrupts this rhythm. Snacking after evening or waiting 3–4 hours after meal helps control glucose levels, but consuming pre-bedtime snacks do not provide the same benefits.

**PROSPERO registration number:**

CRD42023431785.

## Introduction

Type 2 diabetes stands out as one of the most relevant diseases worldwide, primarily due to its complications. According to data from the World Health Organization estimate that around 415 million people worldwide suffer this disease [1]. Type 2 diabetes is a complex metabolic disorder characterized by chronic hyperglycemia resulting from insulin resistance and impaired insulin secretion. There is a variety of factors both intrinsic and extrinsic that participate in its genesis. To understand the complexity of pathological conditions, Sterling & Eyer introduced the concept of allostasis as a process of physiological control that help a biological system to anticipate need [2]. Thus, allostasis describes the body’s ability to adjust to stressful environments through various physiological processes [3] which has been studied and becomes especially relevant in the context of diabetes [4]. Patients undergo persistent physiological stress due to dysregulation in insulin and glucose metabolism, leading to adaptations in hormonal and neural systems aimed at restoring balance [5]. Irregular eating patterns or frequent snacking can significantly impact the body’s allostatic load, posing challenges to glucose regulation and potentially contributing to insulin resistance. Prioritizing changes lifestyle holds particular significance in effectively managing diabetes. Studies have shown that after 4 months of adequate nutritional education, both the dietary intake and lifestyle of patients significantly improve [6]; also, lifestyle adjustments can reduce the incidence of type 2 diabetes (RR = 0.53 (95% CI: 0.41, 0.67)), p < 0.001 according to data from a meta-analysis [7].

In the latest consensus of the "Standards of Care for Diabetes 2023 by the American Diabetes Association", nutritional management takes center stage as an essential component of the comprehensive treatment of patients with type 2 diabetes because it facilitates optimal glycemic control and, subsequently, reducing the risk of chronic complications [8]. There are various nutritional strategies that may be beneficial for patients with type 2 diabetes, addressing factors such as the frequency of meals per day, daily caloric intake, the duration of feeding windows or fasting periods, however, various aspects that influence dietary choices in individuals, including food availability, hunger, satiety, habits, and convenience [9]. Therefore, the following systematic review aims to analyze and assess the relevance of different nutritional strategies in the type 2 diabetes subject focused on the meal frequency, without modifications in the food content, along with the following: time-restricted feeding, role of breakfast, snacks, dinner.

The insights derived from this research hold great potential for tailored dietary recommendations for individuals with type 2 diabetes, enhancing their understanding of meal planning, and potentially improving long-term health outcomes.

## Methods

### Search strategy

The present Systematic Review was performed strictly adhering to the Preferred Reporting Items for Systematic Reviews and Meta-Analyses (PRISMA) guidelines.

A comprehensive literature search related to meal frequency in patients with type 2 diabetes were scanned across the following databases: PubMed, Embase, Web of Science, Cochrane Library, and Google Scholar. The search was made with the following terms (“Diabetes Mellitus” OR “Type 2 Diabetes” OR “DMT2” OR “T2D”) AND (“Meal frequency” OR “Breakfast skipping” OR “Snacks” OR “Chrononutrition” OR “Time-restricted Feeding”) NOT (“risk”) [TI]. From the last 10 years (2013–2023), limited to literature in English. This search was done on July 10th, 2023.

Two authors (RPG and AIC) independently screened titles and abstracts of the articles for eligibility; any disparities between the authors were resolved with the assistance of a third author.

### Study eligibility criteria

To be included in the analysis, studies were selected according to: (I) study design: Observational study, Randomized Controlled Trial or Cross-sectional; (II) humans; (III) Including males and/or females diagnosed with type 2 diabetes older than 19 years old; (IV) if the studies reported any changes in glycemic index or anthropometric measures; (V) published from 2013 to 2023 (the last 10 years). Studies were excluded according to the following criteria: (I) review articles, case reports, systematic review, and meta-analysis, letters, guide, abstracts; (II) patients with type 1 diabetes, risk for type 2 diabetes.

The following question was considered to conduct this systematic review:

What are the strategies regarding meal frequency that have shown to improve the outcomes of subjects with type 2 diabetes?

- Participants—subjects with type 2 diabetes
- Intervention—strategies in meal frequency
- Comparator—Higher number vs fewer number of meals during the day
- Outcome—Improvement of the biochemical parameters of these subjects.

### Data extraction

Information extracted from each study were: first author and publication year, study design, sample size, intervention details like dietary pattern, follow-up, baseline characteristics of the patients, and the outcomes. The measure of the effect was reported with p-value or Odds Ratio (OR) and their 95% Confidence Interval (CI).

The major outcome was glycemic control, including the variation in glycated hemoglobin (HbA1c), weight, and fasting plasma glucose concentration.

### Registration of the protocol

The present review was registered in International prospective register of systematic reviews (PROSPERO) in June 2023, with the registration number: CRD42023431785. This milestone highlights the commitment to transparency and rigor in the research process. PROSPERO registration not only ensures that the review adheres to established guidelines but also facilitates better dissemination and accessibility of the review’s findings.

### Risk of bias assessment and data synthesis

The risk of bias in included studies was assessed by applying the revised Cochrane Risk of Bias tool for randomized trials (RoB 2), and the Risk of Bias in Non-randomized Studies—of Interventions (ROBINS-I).

### Grading of the evidence

The Grading of Recommendations, Assessment, Development, and Evaluations (GRADE) was used to assess the certainty of the evidence, it was graded as high, moderate, low, or very low.

## Results

**Fig 1:**
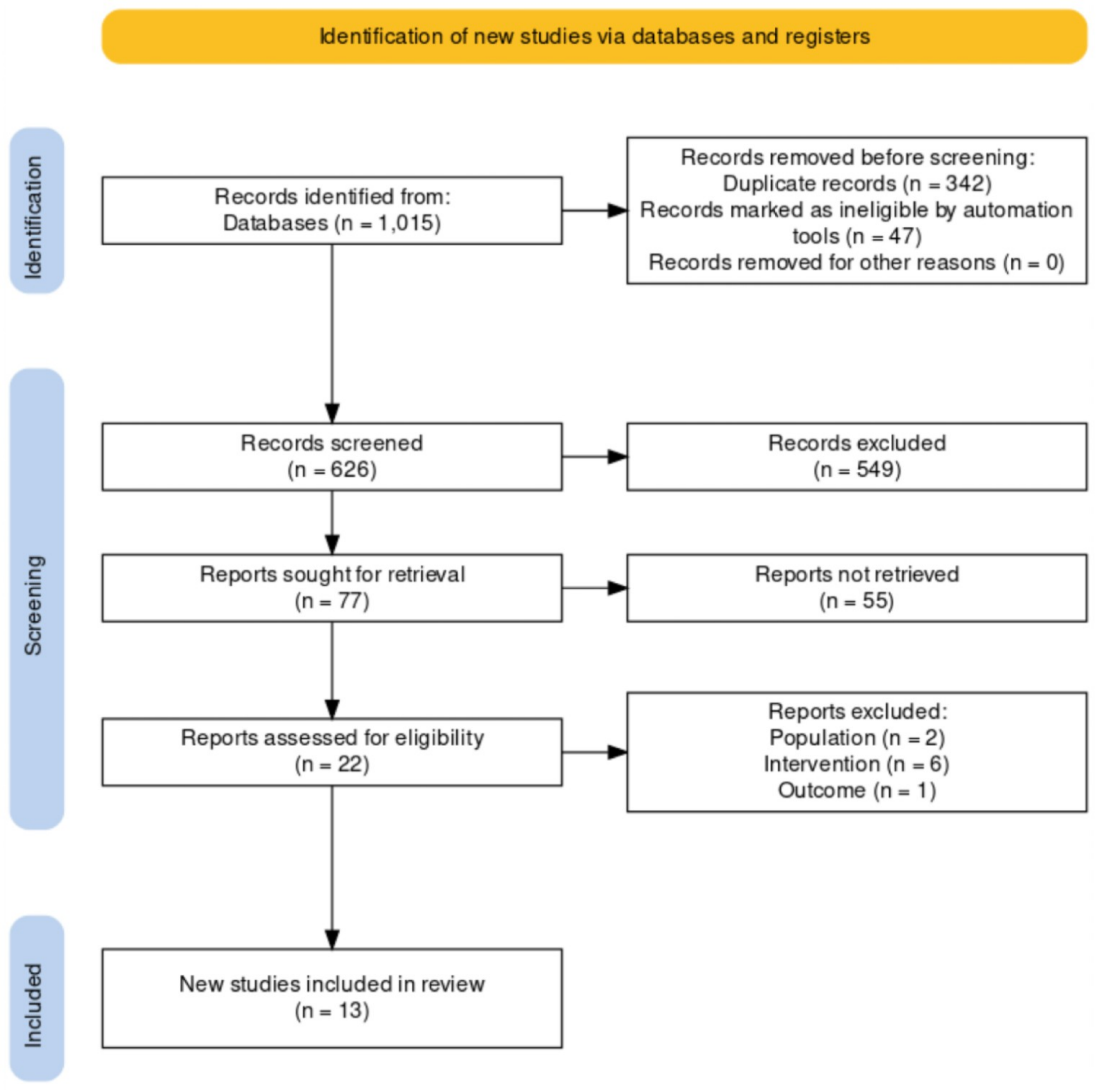
PRISMA flow diagram. Flow diagram of the literature search strategy.

A total of 1015 of potentially relevant articles were identified, of which 389 titles were removed for either being duplicate or not meeting inclusion criteria. Out of the 626 articles remaining were scanned based on the abstract and 77 were sought for retrieval. After a full- text review, 11 randomized trials, 2 non- randomized trials, matched the inclusion criteria and were comprised in the present systematic review (total of 13). The flowchart of study selection is presented in (Fig 1) [10], and the general characteristics of the studies are presented in Table 1.

**Table 1:** Summary of included studies.

REFERENCE	TYPE OF STUDY	N	DURATION	INTERVENTION	MAIN RESULTS
Kahleova et al. [11]	Randomized, crossover	54	24 weeks	Hypoenergetic diet: A6 (6 meals/d) vs B2 (2 meals/d)	BW **↓** (A6: −2.3 vs B2: −3.7 kg; p<0.001). FPG **↓** (A6: −0.47vs B2: −0.68 mmol/l; p = 0.004)
Belinova L et al. [12]	Randomized, crossover, single center	54	24 weeks	Hypoenergetic diet: A6 (6 meals/d) vs B2 (2 meals/d)	Fasting GIP **↓** (A6: -3.36 vs B2: -3.1 pg/mL; p = 0.83) Fasting Leptin **↓** (A6: -1308 vs B2: -1862 pg/mL; p = 0.37) Fasting Ghrelin **↓** (A6: -5.42pg/ml vs B2: 4.5pg/ml; p = 0.023)
Papakonstantinou E. et al. [13]	Randomized Crossover study	47 (12 T2D)	24 weeks	3 vs 6 meals/day	↓ HbA1c and plasma glucose at 120 min post-OGTT with 6 vs 3 meals
Salehil M. et al. [15]	Randomized	84	3 moths	6 meals/day vs 3 meals and 2 snacks previous meal	The 6 meals regimen showed ↓ BW (p = 0.04), HbA1c (p = 0.04), two-hour post-prandial glucose (p = 0.03) and insulin (p = 0.01).
Obermayer A, et al. [16]	Randomized, open-label, controlled trial, single center.	46	12 weeks	IF (18 h) vs CON	HbA1c: **↓**IF (7.3 [12.0]) vs **↑**CON (0.1 [6.1] mmol/mol); p = 0.012 Mean time in range: **↑**IF (68.0 [20.2]) vs CON (56.6 [16.0]%); p = 0.031 BW: **↓**IF (-4.77 [4.99] kg) vs CON (+0.27 [1.34] kg); p< 0.001
Andriessen C et al. [17]	Randomized, crossover, single center	14	10 weeks	TRE (10 h) vs CON (14 h)	FPG **↓** (TRE: 7.6 [0.4] vs CON: 8.6 [0.4] mmol/l; p = 0.03) BW: **↓** TRE (−1.0±0.3 kg, p<0.01) vs CON (−0.3±0.3 kg, p = 0.22)
Che et al. [18]	Randomized controlled trial	104	14 weeks	TRF (10 h) vs CON	HbA1c **↓** (TRF: − 1.54% [0.19] vs. CON: − 0.66% [0.16] mmol/L; p < 0.001) FPG **↓** (TRF: − 1.47 [0.25] vs. CON: − 0.78 [0.21] mmol/L; p < 0.001), BMI **↓** (TRF: − 1.64 [0.38] vs. CON: 0.42 [0.24] kg/m^2^; p < 0.001)
Jakubowicz D et al. [20]	Randomized, open-label, crossover-within-subject clinical trial	18	4 weeks	YesB (breakfast, lunch, and dinner) vs NoB (Lunch and dinner)	YesB AUC_0–180_ for plasma glucose (↑36.8%), FFA (↑ 41.1%), and glucagon (↑14.8%) NoB day AUC_0–180_ for Insulin (**↓**17%) and iGLP-1(↓ 19%)
Abbie E. et al. [21]	Cross-over, randomized	15	3 days	Snack prior to bedtime (egg or yogurt) vs no snack	↓ FPG (p = 0.04), insulin (p = 0.04) and nocturnal glucose (p = 0.02) for patients that had an egg as bedtime snack compared to a yogurt bedtime snack.
Imai S et al. [22]	Randomized open-label, crossover, within-patient clinical trial	17	4 days	Participants wore glucose monitors, ate identical meals, and had snacks at varying times.	↓ SD of glucose, CV, MAGE, MAX and LAGE values in those consuming snacks at 15:30 h than in those who had their snacks at 12:30 h
Imai S. et al. [23]	Randomized, open-label, cross-over, within-patient clinical trial	16	5 days	3 meals/day and on the 2^nd^, and 4^th^ day have either dinner at one time or divided dinner	MPG: greater in the patients that had dinner at one time (8.72 [0.45] mmol/L) vs those who had it divided (8.11 [0.35] mmol/L), p<0.01. Pre-dinner plasma glucose was higher in patients that had dinner divided (7.91 [0.43] vs 6.45 [0.42] mmol/L, p<0.01)
Ashtekar S. et al. [14]	Nonequivalent comparison group quasi‐experimental study.	321	7 months	2 meals/day + workout (2-OMEX) vs control group (≥3 meals/day)	The 2‐OMEX intervention ↓ HbA1c -0.94 gm% (95%CI: ‐1.60 to –0.56) ↓ BW with 2‐OMEX (F: -1.90 kg and M: -1.82 kg) p = 0.562
Parr EB et al. [19]	Pre-post, non-randomized	19	6 weeks	TRE intervention limiting all eating occasions to between 10:00 and 19:00 h	HbA1c (**↓** in TRE -0.2 [0.4]%; p = 0.053) FPG (**↓** in TRE: 8.1 [1.8] vs Habitual Period: 8.4 [2.3]; p = 0.29) Insulin (**↑** in TRE: 17.7 [25.2] vs Habitual Period: 15.0 [15.2], p = 0.09)

Data are expressed as mean (standard deviation) unless otherwise indicated (percentage or p-value). Abbreviations used: N: sample size, ↑: Increase, ↓: decrease, BW: body weight, BMI: body mass index, FPG: fasting plasma glucose, HbA1c: glycated hemoglobin, OGTT: oral glucose tolerance test, CON: control group, TRF: time restricted feeding, TRE: time restricted eating, AUC: area under the curve, FFA: free fatty acids, iGLP-1: intact glucagon-like peptide-1, SD: standard deviation, CV: coefficient of variation, MAGE: mean amplitude of glycemic excursion, MAX: maximum glucose level, LAGE: large amplitude of glycemic excursion, MPG: mean plasma glucose, BS: breakfast skipper, BE: breakfast eaters.

**Fig 2:**
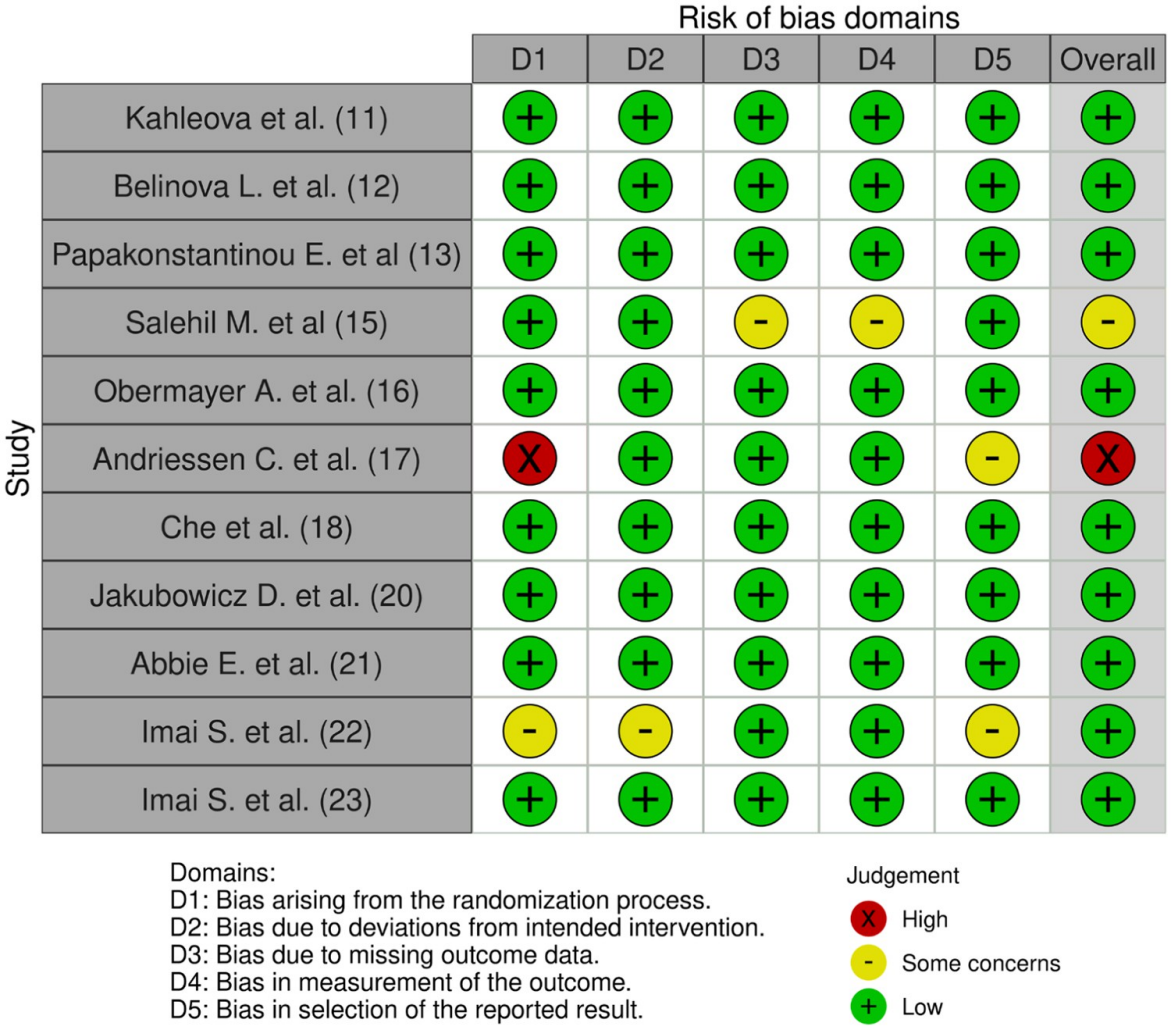
Assessment of the risk of bias for randomized trials. Authors’ judgments about each risk of bias item for every included study in this review.

**Fig 3:**
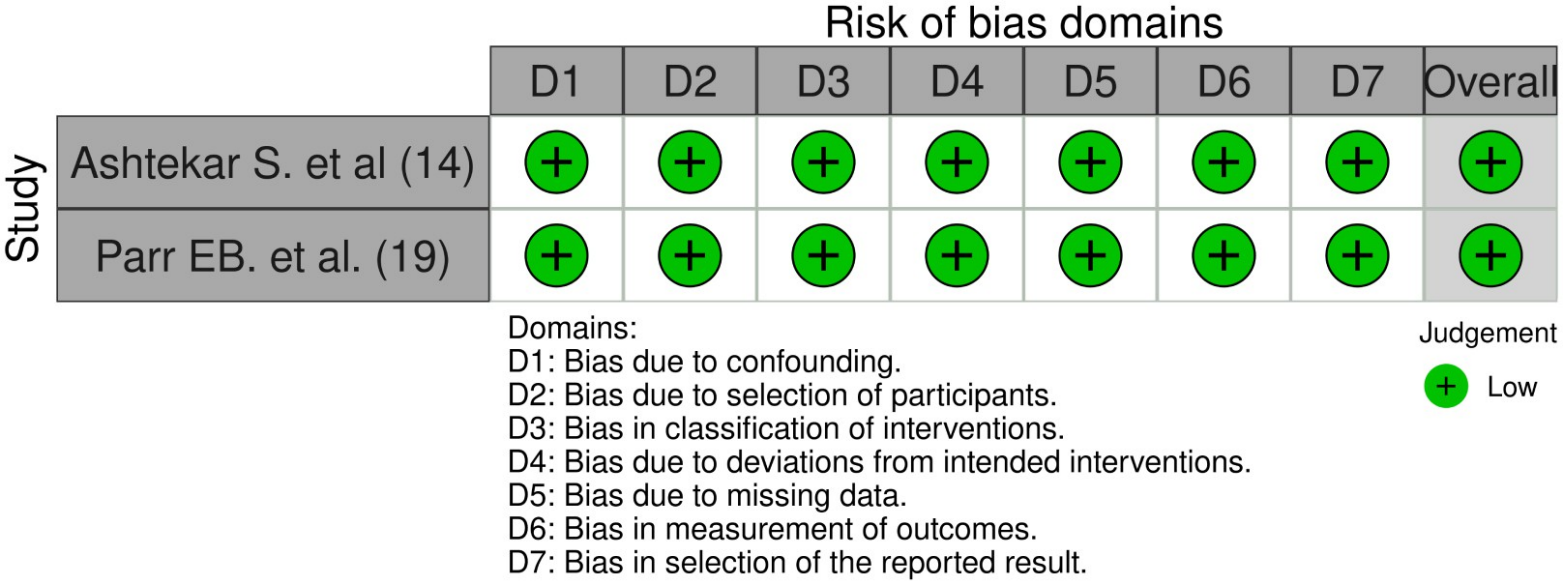
Assessment of the risk of bias for non- randomized trials. Authors’ judgments about each risk of bias item for every included study in this review.

In the comprehensive evaluation of research methodologies, a meticulous assessment of the risk of bias is paramount. For the 11 randomized trials under scrutiny, a rigorous examination of potential biases was conducted, randomization process, deviations from intended intervention, missing outcome data, measurement of the outcome, the reported result. Only 1 of them had high risk of bias due to allocation in the randomization process; the rest had a low risk of bias (Fig 2). Similarly, for the 2 non-randomized trials included in the analysis, an equally thorough assessment of bias was undertaken. Given the inherent differences in study design compared to randomized trials, the evaluation criteria were adjusted accordingly. Factors like selection bias, confounding variables, and outcome assessment methods were closely examined. All the studies resulted in a low risk of bias (Fig 3).

### Meal frequency

A pioneering study to analyze the impact of the number of meals study was conducted by Kahleova H et. al., subjects with type 2 diabetes were randomized into two groups: one consumed a 2-meals/day diet (breakfast and lunch), while the other followed a 6-meal regimen (3 main meals and 3 small snacks). This study showed significant results, indicating reductions in body weight (-3.4 vs -2.0 kg, p<0.001), fasting plasma glucose (-0.78 vs -0.47 mmol/l, p = 0.004), C-peptide (-0.14 vs -0.049 nmol/l, p<0.001) [11]. Furthermore, Belinova L, et. al., observed that in the 2-meals diet, the values of fasting plasma levels of ghrelin were increased in comparison with the 6-meals diet (-5.42 vs 4.5 pg/ml, p = 0.023) [12]. In a randomized crossover study, Papakonstantinou E. et al., compared 3 meals per day vs 6 meals in patients with type 2 diabetes and patients with impaired glucose tolerance during 24 weeks, they came up with the result that patients with T2D benefit from eating 6 times a day, their HbA1c and plasma glucose at 120 min post-OGTT were (p < 0.001 vs p = 0.02, respectively), concluding that consuming six smaller meals has the potential to reduce glucose fluctuations, thereby mitigating the oxidative stress and damage to b-cells that can occur [13].

Ashtekar S. et al, associated that 2 meals/day + a daily walking/aerobic workout for 45 min at least 6 days per week (2-OMEX group) show decrease of HbA1c -0.94 gm% (95%CI: ‐1.60 to –0.56) and weight loss difference of 0.96 kg (p = 0.595) compared with the control group (≥3 meals/day without workout intervention) [14]. Another study demonstrated that a 6 meals/day led to weight lost compared to 3 large meals and 2 snacks previous the meal (p = 0.04) and there was reduction in HbA1c (p = 0.04), two-hour post-prandial glucose (p = 0.03) and insulin (p = 0.01) [15].

### Time-restricted feeding

Time restricted feeding is a dietary strategy that has gained great importance in recent years, especially in patients with type 2 diabetes, especially as it promotes better glycemic control. In a randomized study where subjects were assigned to either an Intermitted Fasting (IF) group or a control group, after a 12-week period, the IF group experienced a decrease of 7.3 [12.0] mmol/mol in HbA1c levels, while the control group exhibited an increase of 0.1 [6.1] mmol/mol (p = 0.012) [16].

Andriessen C et al., compared a 14-hr. vs 10-hr. feeding window for 3 weeks in subjects with type 2 diabetes; the results show that the 10-hr. feeding window significantly decreased fasting plasma glucose levels (8.9 [0.5] vs 8.0 [0.3] mmol/l, p = 0.04). Triglyceride, and insulin values also decreased in both regimens, although no significant changes were observed [17]. In another study comparing a 10-hour TRF against a control group that maintained their normal dietary habits, subjects who underwent TRF exhibited significant reduction in HbA1c (-1.54 [0.19]% vs -0.66 [0.16]%; p<0.001), fasting plasma glucose (-1.47 [0.25] mmol/l vs -0.78 [0.21] mmol/l; p<0. 001) and HOMA-IR (-0.51 [0.08] vs -0.12 [0.06]; p = 0.02) and increase in HOMA B (0.73 [0.21]% vs 0.27 [0.10]%; p = 0.005); additionally, significant improvements in cardiovascular risk markers such as total cholesterol and triglycerides were observed [18]. The last study we reviewed was from Parr EB. et al., analyzed the impact of a 9-hour TRF performed only 5 days a week over a 4-week period. The findings indicated a non-significant reduction in HbA1c (−0.2 [0.4]%; p = 0.053), and no variation were identified in fasting glucose (p = 0.29), insulin (p = 0.9), or total cholesterol (p = 0.16) [19].

### Role of breakfast

A debatable subject in nutritional research is the role of breakfast, both in terms of eating and skipping. Jakubowicz D et al., in a cross-over study with a washout period of 2–4 weeks, tested the effect of skipping breakfast on postprandial hormones after lunch, and dinner in subjects with type 2 diabetes who were divided into two groups. The first one receiving breakfast, lunch, and dinner (YesB), while the other group received lunch and dinner (NoB); they demonstrated a higher area under the curve (AUC) for postprandial concentrations of glucose (16.5%), insulin (45%), C-peptide (50%), intact glucagon-like peptide-1 (iGLP-1) (33%) in the YesB group compared to the NoB group (p< 0.0001) [20].

### Snacks

The term snack should be understood as a food outside an established meal plan (e.g. breakfast, lunch and dinner). Abbie et al. conducted a study involving patients with type 2 diabetes who followed a standardized isoenergetic diet during the day. The participants were divided into two groups: one consumed a snack prior to bedtime, while the other group did not, they found no significant difference in fasting plasma glucose (7.9 [0.3] mmol/L) or fasting plasma insulin (p> 0.25), between the two groups, similarly, no difference was found between the ones that had the snack with either an egg (7.6 [0.2] mmol/L) or a yogurt (8.2 [0.3] mmol/L), (p>0.36) [21]. Imai S et al., investigated the benefits of consuming an evening snack of 75 calories in addition to a 3-course diet (breakfast, lunch, and dinner), this snack was to be eaten either right after the lunch or 3–4 hours after the meal. The study revealed a significant reduction in the mean glycemic excursion amplitude (6.9 vs 5.19 mmol/L, p = 0.001), as well as a decrease in postprandial serum glucose after dinner in the subjects who consumed the snack during this study [22].

### Role of dinner

Imai S. et al., showed that subjects who consumed dinner in one sitting exhibited significantly higher values in the incremental AUC (644 [156] vs 147 [63] mmol/L x min, p< 0.01), and the incremental glucose peak after dinner (6.78 [0.79] vs 3.09 [0.62] mmol/L, p< 0.01) compared to those who divided their dinner [23].

## Discussion

The role of nutrition in individuals with type 2 diabetes is a vast and multifaceted subject. Numerous studies delve into various aspects, including the number of meals per day, meal frequency, nutritional intake, and the distribution of nutrients within each meal. These investigations contribute to a comprehensive understanding of how dietary factors impact the management and well-being of individuals living with type 2 diabetes. Therefore, this analysis aims to systematically review the evidence regarding the impact of meal frequency strategies on glycemic control in individuals with type 2 diabetic. One of the most extensively studied dietary intervention that has gained recent attention is meal frequency. There appears to be a correlation between the frequency of daily meals and both chronic diseases and positive changes in biochemical markers related to type 2 diabetes. Based on the studies reviewed in this work, it is hypothesized that consuming fewer meals during the day is associated with improvements in fasting plasma glucose and C-peptide in subjects with type 2 diabetes. In addition to the benefit of frequency-based feeding control in the control of blood glucose excursions, it has been demonstrated that reduced meal frequency is also considered beneficial for weight loss.

Lately, there has been a heightened emphasis on the impact of meal synchrony, along with the importance of maintaining a proper and consistent schedule for food consumption. Recent strategies underscore the fundamental role of the circadian cycle in orchestrating these dietary interventions. Circadian clocks regulate biological processes through day/night cycle [24]. They modulate both insulin, and glucagon by controlling production and secretion at the cellular level, as well as signaling in the central nervous system [25]. Chrono-nutrition involves coordinating meals with the circadian rhythm, recognizing that in addition to the quality and quantity of food, the timing of feeding plays a critical role in health.

While the central objective of this study is to illustrate the significance of nutritional strategies contingent upon the daily quantity of food intake, without delving into the caloric content of individual foods, it is inevitable to address the relationship of diet and the moments in which it is carried out with the physiopathogenesis of type 2 diabetes. Consuming food at anticipated times triggers metabolic pathways that help maintain nutritional homeostasis and provide feedback to the circadian cycle, leading to beta cell dysfunction and insulin sensitivity [9]. Individuals with type 2 diabetes show increased insulin sensitivity in the evening and greater glycemic variations in the morning than in the afternoon; irregular eating patterns in these individuals results in desynchronization, reducing insulin sensitivity levels and causing postprandial glucose intolerance. [26]. It has been proposed that modern lifestyle habits, such as abnormal sleep patterns, jet lag, inadequate sun exposure, and night-time work shifts contribute to circadian cycle disruption [25]. Circadian cycle asynchrony contributes to the pathophysiology of type 2 diabetes, as down-regulation of circadian cycle genes is associated with insulin resistance, decreased insulin secretion, increased postprandial glucose, and elevated HbA1c levels [27].

Intermittent fasting has emerged as an adjuvant strategy in the treatment of several diseases, including diabetes, obesity, cancer, and neurodegeneration [28]. Intermittent fasting (IF) has been proposed as a dietary strategy for glycemic control and weight loss in obese and with type 2 diabetes. There are multiple types of IF such as alternate-day fasting, full-day fasting and time-restricted feeding (TRF) [29]. TRF shortens the feeding window, typically occurring within 12 to 14 hours, to a period of 6 to 10 hours during the active phase of the day, thereby extending the fasting period without altering the quantity or quality of food consumed. It is suggested that the feeding window align with the natural rhythms of the circadian cycle, results in reduced body weight, improved blood pressure, enhanced glucose tolerance [9], insulin sensitivity and an overall improvement of cardiometabolic risk. Furthermore, the flexibility of TRF schedules makes them feasible for individuals to follow [19]. Studies have shown that subjects following a TRF-based diet tend to spontaneously decrease their caloric intake by 7 to 22% when eating ad libitum, demonstrating potential benefits related to the food content independent of the feeding frequency [30].

The role of breakfast in the diets of both subjects with and without diabetes, has always been a substantial topic of discussion when considering the adoption of healthy eating habits. Breakfast consumption plays a critical role in achieving metabolic control in individuals with type 2 diabetes since its omission, a common behavior in the population, disrupts the expression of circadian cycle genes, leading to postprandial hyperglycemia, insulin deficiency, and poor GLP-1 response to subsequent food consumption [27]. This is explained by the second meal or Straub-Traugott phenomenon, which describes how the first meal optimizes beta cells responsiveness of to the second meal, induced by the first meal. In other words, there is a potentiation of both the first and second phases of insulin release upon prior exposure to glucose [31, 32]. Clearly, skipping breakfast has a detrimental effect on the glycemic control of individuals with type 2 diabetes.

On the other hand, incorporating snacks alongside main meals throughout the day to enhance glycemic control has rarely been adopted as a dietary strategy. It has been proposed that consuming a pre-bedtime snack may improve glycemic control by mitigating the effects of fasting hyperglycemia, reducing the overnight fasting window, and decreasing hepatic demands for gluconeogenesis in individuals with type 2 diabetes and those with prediabetes [33]. Several studies have addressed the impact of eating dinner after 21:00, demonstrating prolonged postprandial hyperglycemia due to reduced diet-induced thermogenesis at the end of the day, which affects glucose tolerance from morning to evening [34, 35].

Regarding the alterations made to the dinner regimen, research indicates that dividing the dinner into two separate occasions has demonstrated improved glycemic control compared to a single intake of an equivalent quantity. This suggests that superior control may be attained by dividing one of the principal meals.

The limitations of this systematic review are firstly, this review only included studies published in English, which may introduce language bias, and potentially exclude relevant studies published in other languages. Secondly, the current number of studies available that evaluate this topic remains limited.

## Conclusion

Individuals with diabetes face challenges in adopting healthy eating habits, particularly within the obesogenic environment caused by current lifestyle and unhealthy dietary patterns, leading to persistence of deleterious eating practices, impacting both health and metabolic control. Reducing meal frequency appears to be an optimal approach since, according to recent studies, the intake of 2 to 3 meals instead of 6 meals per day promotes weight loss and glycemic control in subjects with type 2 diabetes. Time Restricted Feeding is a dietary strategy that does not restrict caloric intake or food composition, it only addresses feeding periodicity by prioritizing intake during the active phase and restricting it to less than 10 hours per day. This approach has been demonstrated benefits in reducing fasting and postprandial glycemic variability as well as weight loss. Skipping breakfast, a common practice influenced by current lifestyle, disrupts the circadian cycle leading to postprandial hyperglycemia, insulin deficiency, increased glycosylated hemoglobin, and weight gain. On the other hand, pre-bedtime snacks have not been shown to benefit diabetic subjects in glycemic control.

When delving into dietary considerations, it’s crucial to extend the conversation beyond the mere frequency of meals and incorporate the principle of energy balance. Individuals managing diabetes must ensure that their calorie intake aligns harmoniously with their energy expenditure, as this equilibrium is pivotal for overall health and blood sugar control. Attaining the right balance becomes particularly essential for effective weight management, a key facet in diabetes care, as excess weight can heighten insulin resistance. Beyond mere caloric calculations, the nutritional quality of food choices also exerts a significant influence on energy balance, underscoring the importance of opting for nutrient-dense foods.

Further research is needed to provide specific recommendation for clinical practice regarding the periodicity of feeding in individuals with type 2 diabetes. However, it is essential to recognize that dietary recommendations focused on feeding frequency deserve the same importance as those that only consider the quality and quantity of food.

## Direction for future research

While existing studies offer valuable insights into the impact of meal timing and frequency on metabolic health, there is a need for more comprehensive and longitudinal investigations. Additionally, investigating cultural and regional variations in dinner practices may contribute to tailoring dietary recommendations for diverse populations.

## Supporting information

S1 File DOIS. (DOCX)

S2 File PRISMA checklist. (PDF)

S3 File PROSPERO registration. (PDF)

S4 File Search terms. (DOCX)
