# Supplementary material for: Meal frequency strategies for the management of type 2 diabetes subjects: A systematic review
Source: PLoS One. 2024 Feb 29;19(2):e0298531. doi: 10.1371/journal.pone.0298531 (PMC10903815; doi:10.1371/journal.pone.0298531)
Supplement: S1 File — (DOCX) [file pone.0298531.s001.docx]

**DOIS**

10.1111/ijcp.12991

10.2174/1573399812666160901094741

10.1177/1043659615597041

10.1016/j.diabres.2014.09.026

10.1007/s12664-016-0694-4

10.1111/dme.12671

10.3390/nu10121834

10.2337/dc14-0991

10.1007/s12325-018-0704-2

10.1016/j.numecd.2017.07.005

10.1371/journal.pmed.1002052

10.1016/j.soard.2015.10.076

10.1111/dom.12810

10.1016/S1262-3636(16)30004-0

10.2147/DMSO.S141235

10.1530/EJE-16-0286

10.4158/EP14540.OR

10.1016/j.jdiacomp.2017.05.010

10.1507/endocrj.EJ14-0194

doi: 10.1177/2040622315608646

10.1080/17446651.2018.1518131

10.2337/dc15-2782

PMID: 31309801

10.1002/dmrr.2525

10.1089/dia.2014.0073

10.1055/s-0042-115175

10.1007/s11892-018-1102-5

10.1111/dme.13259

10.1089/jwh.2017.6788

PMID: 25799651

10.1097/MD.0000000000010981

10.1016/j.jdiacomp.2013.10.003

10.1186/s40064-016-1932-z

10.1016/j.metabol.2015.06.004

10.1111/nmo.13958.

PMID: 25242841

10.1017/S000711451900076X

PMID: 26400122

10.1371/journal.pone.0191584

10.3945/an.115.010397

10.2147/DMSO.S209320

PMID: 31760400

10.1007/s13105-017-0582-0

10.1016/j.jand.2014.11.018

10.3390/nu8110687

10.1080/07420528.2017.1279624

PMID: 31022339

10.1111/dom.13060

10.1002/ptr.5796

10.1139/apnm-2016-0467

10.1371/journal.pone.0216534

10.3390/nu9040352

10.3390/nu9020152

10.1186/1475-2840-13-104

10.1017/S0007114518000703

10.1186/s12872-016-0380-6

10.1016/S2213-8587(13)70090-1

10.1016/j.ypmed.2014.11.025

10.1111/j.2047-6310.2013.00147.x

10.1017/S0007114515005346

10.3389/fnut.2017.00033

10.1152/ajpendo.00593.2013

10.1111/dom.13016

10.1155/2016/9759241

10.1016/j.jdiacomp.2017.06.008

10.1017/S0007114516003214

10.1016/j.numecd.2015.06.011

10.3390/nu7053449

10.1185/03007995.2014.933098

10.1111/dme.13081

10.1002/jcph.1321

10.2337/dc13-3007

10.1530/EJE-17-0446

10.1016/j.ghir.2015.08.002

10.3945/ajcn.115.120170

10.1080/07315724.2018.1545611

10.1111/cen.13977

10.1016/j.jsams.2019.01.017

10.3892/etm.2019.7376

10.1371/journal.pone.0215636

10.1210/jc.2018-01809

10.1007/s11606-018-4812-0

10.1007/s11606-018-4716-z

10.1111/dme.13829

10.1111/pedi.12790

PMID: 32060194

PMID: 30755550

10.1016/j.diabet.2018.05.008

10.1016/j.jneb.2017.08.005

10.2147/AHMT.S178746

10.1177/1049732318784906

10.1038/s41387-018-0054-9

10.1007/s12079-017-0427-1

10.1016/j.appet.2018.03.020

10.1111/1753-0407.12658

10.1371/journal.pbio.2005143

10.2337/db17-1226

10.1210/jc.2018-00496

10.1507/endocrj.EJ18-0088

10.1136/bmj.k2234

10.1016/j.diabet.2018.03.008

10.1007/s13300-018-0419-z

10.3122/jabfm.2018.03.170280

10.1111/dom.13214

10.1507/endocrj.EJ17-0386

10.1186/s13104-018-3138-7

10.1038/s41598-017-18913-7

10.1371/journal.pone.0191114

10.1007/s11695-017-2804-5

10.1177/0145721717730646

10.1186/s41043-017-0115-z

10.1016/j.phrs.2017.09.005

10.1017/S1368980017001860

10.1080/07435800.2017.1294602

10.4239/wjd.v8.i10.455

10.1111/dom.12955

10.1111/dme.13390

10.1507/endocrj.EJ17-0020

10.1111/1753-0407.12456

10.1016/j.appet.2017.03.005

10.1016/j.psychres.2017.02.049

PMID: 29293296

10.1371/journal.pone.0174820

10.1177/0748730417693480

10.1016/j.metabol.2017.01.023

10.1111/1753-0407.12426

10.1002/osp4.92

10.1111/dom.12826

10.1007/s11524-016-0129-7

10.1016/j.diabres.2016.12.011

10.1111/dom.12800

10.1002/dmrr.2816

10.1111/dom.12748

10.1089/dia.2016.0176

10.1016/j.diabres.2016.09.014

10.4067/S0034-98872016001000002

10.1161/ATVBAHA.116.307930

10.1136/bmjdrc-2016-000228

10.1016/j.soard.2016.02.007

10.1007/s00592-016-0838-0

10.4103/2230-8210.183472

10.1089/dia.2016.0010

10.1185/03007995.2016.1161609

10.4103/2230-8210.180003

10.1080/07315724.2015.1046197

10.1186/s12933-016-0374-9

10.1089/dia.2015.0290

10.1016/j.diabres.2015.12.004

10.1089/dia.2015.0082

10.1155/2016/7987395

10.1016/j.nut.2015.07.011

10.1016/j.abb.2015.08.003

10.1017/S0007114515003645

PMID: 27045156

10.2337/dc15-0160

10.4158/EP14498.OR

10.1111/dom.12528

10.3945/ajcn.115.107599

10.1016/j.metabol.2015.04.008

10.1590/S0080-623420150000400012

10.1016/j.clnu.2014.08.016

10.4158/EP15675.OR

10.1177/1932296815569882

10.1016/j.jand.2014.11.003

10.2337/dc14-1796

10.1007/s10903-013-9948-8

PMID: 25366984

10.1186/s12933-015-0181-8

10.1007/s40618-014-0170-x

10.1186/s12933-014-0169-9

10.2169/internalmedicine.54.3498

10.6133/apjcn.2015.24.3.03

PMID: 26400122

10.1155/2015/236234

10.1177/1932296814555400

10.1371/journal.pone.0114135

10.1016/j.diabres.2014.09.044

10.1016/j.diabres.2014.09.040

10.1007/s00104-014-2799-6

10.2337/dc14-0876

10.1111/dom.12303

10.1055/s-0034-1370963

10.1185/03007995.2014.929097

10.1111/dme.12434

10.1016/j.jdiacomp.2014.03.014

10.1185/03007995.2014.912983

10.1185/03007995.2014.901943

10.1186/1743-7075-11-28

10.1016/j.jand.2014.02.004

10.1007/s00125-014-3216-x

10.1016/j.diabres.2013.12.047

10.1007/s00592-012-0420-3

PMID: 25686619

PMID: 30418612

10.3390/nu15010085

10.1007/s13300-022-01261-9

10.1155/2022/7245223

10.3390/nu13051558

10.1016/j.nut.2019.110639

10.1017/S0007114519001235

10.1093/ajcn/nqy346

10.4239/wjd.v9.i11.190

10.2147/DMSO.S176749

10.1016/j.jcjd.2017.07.007

10.1507/endocrj.EJ17-0414

10.1136/bmjopen-2017-019589

10.2337/dc16-2753

10.19082/5179

PMID: 29927569

10.1186/s12889-016-3527-6

10.1016/j.jcjd.2015.08.015

10.1016/j.dsx.2015.09.022

10.2337/dc15-0761

10.1016/j.orcp.2013.01.001

10.1155/2014/253581

10.3390/nu14040823

10.7759/cureus.15853

10.3109/07420528.2013.821614

10.2337/dc12-2697

10.1007/s13410-019-00734-1

10.1038/sj.ejcn.1602089

10.1038/nutd.2013.3

10.2337/dc23-1267

10.1007/s00125-023-06008-0

10.1016/j.ejps.2023.106644

10.1186/s12913-023-10293-1

10.1371/journal.pone.0289289

10.1136/bmjmed-2023-000664

10.1017/S1368980023001106

10.3389/fendo.2023.1283626

10.3390/nu15204369

10.1093/ije/dyad081

10.1002/jbio.202300083

10.1177/19322968231197205

10.1093/tbm/ibad029

10.1007/s40618-023-02187-0

10.3389/fnut.2023.1219381

10.1016/j.cct.2023.107307

10.1016/j.tjnut.2023.07.011

10.1371/journal.pone.0290261

10.1111/jhn.13145

10.1371/journal.pone.0282401

10.1186/s12916-023-02938-z

10.2337/dc22-2297

10.3390/nu15122791

10.3390/nu15122657

10.1117/1.JBO.28.6.065002

10.1007/s40200-022-01153-6

10.1016/j.smrv.2023.101788

10.1016/j.jneb.2023.02.010

10.3390/nu15071762

10.1146/annurev-publhealth-071521-121621

10.1007/s13300-023-01379-4

10.1055/a-2018-4299

10.1111/ijpo.13000

10.3390/nu15061385

10.3390/nu15061348

10.1016/j.cmet.2023.02.003

10.5455/msm.2023.35.13-17

10.7759/cureus.35650

10.1002/oby.23678

10.7759/cureus.35486

10.3389/fendo.2023.1051592

10.3390/nu15030702

10.1038/s41467-023-36013-1

10.3389/fendo.2022.1094954

10.4103/ijcm.ijcm_248_22

10.1016/j.cct.2022.107039

10.1371/journal.pone.0279466

10.3390/ijerph20010599

10.1093/ajcn/nqac258

10.1093/ajcn/nqac207

10.3390/jpm12111881

10.1111/jdi.13876

10.1111/1747-0080.12741

10.1038/s41430-022-01160-z

10.3390/nu14204290

10.1001/jamanetworkopen.2022.36123

10.1038/s41366-022-01193-1

10.1002/dmrr.3558

10.1017/jns.2022.56

10.1001/jamanetworkopen.2022.33760

10.1093/ajcn/nqac087

10.1017/S0007114521003226

10.1017/S0007114521002944

10.3389/fnut.2022.925870

10.3389/fpubh.2022.912816

10.3390/nu14132646

10.3390/ijerph19137714

10.1007/s00431-022-04445-4

10.1177/1742395320959434

10.1371/journal.pone.0267977

10.4103/ijem.ijem_79_22

10.3390/nu14091810

10.1371/journal.pmed.1003970

10.3390/nu14091719

10.1038/s41387-022-00188-1

10.1590/S0004-2803.202202000-42

10.25122/jml-2021-0259

10.1016/j.jcjd.2021.09.005

PMID: 35443344

PMID: 35399945

10.1007/s00592-021-01809-4

10.1002/dmrr.3504

10.1136/bmjdrc-2021-002534

10.1002/oby.23340

10.1210/clinem/dgab655

10.3390/nu14020376

10.3390/nu14020372

10.3390/nu14020366

10.1007/s00125-021-05577-2

10.1016/j.jdiacomp.2021.108028

10.3390/nu13124492

10.1093/tbm/ibab113

10.1007/s42000-021-00314-1

10.3389/fnut.2021.765543

10.1097/MD.0000000000027629

10.1002/fsn3.2570

10.1016/j.dsx.2021.102317

10.1111/1753-0407.13185

10.3390/ijerph182111056

10.2196/32298

10.1111/dme.14657

10.1186/s12933-021-01385-5

10.4081/jphr.2021.2283

10.3390/nu13103344

10.1136/bmjdrc-2021-002350

10.1007/s12020-021-02810-1

10.1016/j.sleep.2021.05.023

10.1136/bmjopen-2020-046183

10.1136/openhrt-2021-001680

10.2337/dc21-0269

10.3390/cimb43020039

10.1210/jendso/bvab067

10.1016/j.jpsychores.2021.110463

10.1111/dom.14346

10.1111/ijpo.12757

10.1016/j.jcjd.2020.09.009

10.1017/S0007114520001944

10.3390/nu13051651

10.1371/journal.pone.0250832

10.1186/s12986-021-00556-1

10.1016/j.dsx.2021.03.032

10.1089/jmf.2020.0082

10.3390/nu13041179

10.3390/medicina57040341

10.3390/nu13041144

10.1136/bmjdrc-2020-002034

10.1016/j.nutres.2020.12.015

10.1111/dme.14515

10.1161/ATVBAHA.120.315446

10.2337/dc20-1567

10.3390/ijerph18030909

10.1111/dom.14216

10.1210/clinem/dgaa653

10.1111/dme.14394

10.1111/nmo.13958

10.1002/osp4.444

10.2337/dc19-2550

10.1007/s00394-020-02212-5

10.1371/journal.pone.0242360

10.1111/dme.14096

10.3389/fendo.2020.584642

10.1024/0300-9831/a000463

10.3390/nu12103013

10.3389/fnut.2020.537049

10.1111/dom.14078

10.3390/nu12092553

10.3390/nu12082327

10.1093/cdn/nzaa126

10.1177/0300060520945885

10.2337/dc19-2232

10.1177/0897190018818908

10.1002/oby.22777

10.1111/dme.14312

10.3390/ijerph17124268

10.1136/bmjdrc-2019-000899

10.1080/07420528.2020.1772810

10.1016/S2213-8587(20)30117-0

10.3390/ijerph17093322

10.1186/s12902-020-0521-x

10.1136/bmjdrc-2020-001244

10.1093/advances/nmz132

10.1371/journal.pone.0230554

10.1016/j.diabres.2020.108076

10.1210/clinem/dgaa070

10.1093/ajcn/nqaa014

10.1007/s00125-019-05083-6

10.2337/dc19-1843

10.1111/dme.14193

10.1111/dme.14191

10.1002/jcph.1549

10.1210/clinem/dgz189

10.1007/s13300-019-00732-w

10.1210/clinem/dgz047

10.1038/s41387-020-0109-6

10.1136/bmjdrc-2019-001162

PMID: 32060194

10.1016/j.jcjd.2019.04.015

10.1007/s11892-020-1291-6

10.1186/s12902-020-0491-z

10.1159/000508843

10.6133/apjcn.202003_29(1).0010

PMID: 31915353

10.1111/dom.13871

10.1111/jdi.13093

10.1186/s12889-019-8030-4

10.2337/db19-0378

10.1017/S1368980019001940

10.1016/j.molmet.2019.08.016

10.1093/ajcn/nqz191

PMID: 31760400

10.3934/publichealth.2019.4.424

10.1136/bmjopen-2018-028076

10.3390/nu11102476

10.1186/s12871-019-0848-x

10.1371/journal.pcbi.1007400

10.1016/j.surg.2019.06.037

10.1007/s11096-019-00877-5

10.3390/nu11092209

10.14814/phy2.14189

10.1007/s00125-019-4906-1

10.2169/internalmedicine.2133-18

10.1002/oby.22522

10.1016/j.diabres.2019.06.007.

10.3390/nu13082748

10.4314/ejhs.v27i3.4

10.3390/nu11030486

10.1016/j.clnu.2020.03.008

10.1016/j.metabol.2017.11.017

10.1016/j.eatbeh.2016.03.001

10.1186/s12889-018-6101-6

10.1016/j.cell.2015.11.001

10.1038/s41591-023-02287-7

10.2337/dc20-0263

10.3389/fnut.2023.1122102

10.1159/00035733

10.1007/s00125-019-4956-4

10.1002/oby.22449

10.1097/MPG.0000000000001733

10.1186/s12889-015-1941-9

10.23736/S2724-6507.20.03369-6

10.2337/dc21-1314

10.3389/fendo.2022.874968

10.1007/s00508-023-02181-9

10.1016/j.ajcnut.2023.02.017

10.3945/an.115.008623

10.3389/fendo.2019.00144

10.3390/nu14030706

10.3389/fendo.2022.875535

10.1038/s41430-021-00940-3

10.1136/bmjopen-2022-063888

10.1097/MD.0000000000032805

10.1016/j.diabres.2021.108941

10.1007/s00125-021-05572-7

10.1210/clinem/dgad273

10.1111/dom.13503

10.1016/j.clnu.2020.10.005

10.2337/dc20-0460

10.1016/j.cct.2021.10651

10.3390/nu12061561

10.1152/ajpendo.00109.2022

10.15605/jafes.036.02.03

10.1016/j.diabet.2018.07.001

10.3390/nu12092502

10.1111/1541-4337.12762

10.2337/dc18-2270

10.1155/2022/4875993

10.1038/s41387-021-00161-4

10.1007/s12020-016-1018-2

10.2337/dc19-1142

10.1017/S1368980014002973

10.1007/s00125-015-3841-z

10.3945/ajcn.116.135343

10.1038/s41430-022-01128-z

10.1038/s41387-019-0093-x

10.1177/0260106018800074

10.1016/j.numecd.2017.05.007

10.1249/MSS.0000000000002165

10.3389/fnut.2023.1216753

10.1016/j.dsx.2019.05.026

10.1186/1475-2891-13-10

10.3945/jn.114.202549

10.1007/s00125-016-4169-z

10.2337/dci19-0061

10.1177/2040622315608646

PMID: 25799651

10.1111/nmo.13958

10.1111/1747-0080.12741

10.1016/j.diabres.2019.06.007

10.1016/j.tjnut.2023.07.011

10.1111/dme.14096

10.1007/s00125-019-05083-6

10.1016/j.diabres.2019.06.007

10.1007/s13300-018-0419-z

10.1007/s00592-016-0838-0

10.1016/j.soard.2015.10.076

10.1155/2016/9759241

10.1016/j.abb.2015.08.003

10.4158/EP14540.OR

PMID: 25366984

10.2169/internalmedicine.54.3498

10.1185/03007995.2014.912983

10.1185/03007995.2014.901943

10.1152/ajpendo.00593.2013

10.1210/jendso/bvab049

10.3390/nu12113228

10.1186/s12986-021-00613-9

10.1007/s00125-022-05752-z

10.2337/dc22-1622

10.1007/s00125-014-3253-5

10.1007/s10529-022-03232-3

10.1093/jn/nxy284

10.3389/fendo.2022.836023

10.1371/journal.pmed.1001703

10.1111/dme.13642

10.1093/jn/nxy194

10.3390/nu13051440

10.1016/j.eclinm.2021.101241

10.1007/s13340-020-00457-3

10.1111/jdi.12852

10.1016/j.athplu.2021.08.006

10.1017/S0007114514001123

10.1186/s12902-018-0297-4

10.2169/internalmedicine.55.7085

10.3945/jn.114.195339

10.2188/jea.JE20140109

10.3390/nu6125740

10.1371/journal.pgph.0000802

10.1186/s13063-023-07691-5

10.1007/s00125-015-3524-9

10.1016/j.diabres.2018.10.010

10.1093/ajcn/nqy019

10.3945/ajcn.116.151332

10.1007/s00125-014-3457-8

10.1371/journal.pone.0079324

10.1186/s12933-016-0398-1

10.1093/ajcn.82.1.69

10.1038/sj.ejcn.1602427

10.1016/j.jnutbio.2017.07.005

10.2337/diacare.22.5.789

10.1079/bjn19970054

10.1517/14656566.2014.939070

10.1016/s1056-8727(97)00073-1

10.3945/jn.115.210617

10.1177/1932296815586425

10.26402/jpp.2018.5.02

10.1016/j.diabres.2021.109003

10.1210/clinem/dgaa926

10.1007/s13105-021-00839-4

10.1136/bcr-2017-221854

10.3390/nu13093179

10.1113/JP281101

10.1055/s-0043-101700

10.1016/j.jad.2021.12.110

10.1111/1753-0407.13329

10.3389/fendo.2022.975468

10.1007/s12325-020-01410-1

10.1136/bmjdrc-2021-002406

10.1016/j.diabet.2017.09.001

10.3390/nu11030607

10.1017/S1368980015000257

10.1038/ejcn.2015.46

10.1007/s11892-004-0019-3

10.1186/1471-2458-13-740

10.1016/j.ajcnut.2023.08.021

10.1371/journal.pmed.1004221

10.3390/nu15092083

10.3390/nu15071787

10.3389/fendo.2023.1113611

10.1186/s12992-023-00910-3

10.3390/nu14183691

10.1007/s00421-019-04261-z

10.1152/japplphysiol.00389.2018

10.1186/1472-6823-15-1

10.1039/c9fo01778j

10.1111/dom.14255

10.1111/jhn.12822

10.1371/journal.pmed.1003409

10.1016/j.appet.2020.104744

10.1136/bmjdrc-2020-001258

10.20960/nh.02590

10.1055/a-0631-8813

10.1136/bmjopen-2018-028544

10.1016/j.clnesp.2018.12.088

10.1002/oby.22406

10.3390/nu12071949

10.3390/nu13041298

10.1159/000512142

10.2196/jmir.5806

10.1016/j.numecd.2019.02.004

10.3390/nu11040766

10.3390/nu10060661

10.1002/dmrr.3188

10.3390/nu13072378

10.1186/s12902-022-00947-2

10.1111/dom.13164

10.3390/nu15204369.

10.1136/bmjdrc-2022-002820

10.1007/s00125-021-05400-y

10.1016/j.ajcnut.2023.04.032

10.1039/d3fo02165c

10.1016/j.pcd.2016.08.005

10.2302/kjm.2016-0016-IR

10.1016/j.nut.2014.06.011

10.20960/nh.04204

10.1002/dmrr.2515

10.1371/journal.pone.0242487

10.1002/dmrr.2519

10.1097/01.JAA.0000546481.02560.4e

10.1111/1747-0080.12513

10.1016/j.diabres.2020.108579

10.1056/NEJMc1312802

10.3390/nu11081905

10.1016/j.jcjd.2013.03.023

10.3177/jnsv.66.114

10.1016/j.sleep.2018.12.014

10.1080/17446651.2019.1554430

10.1007/s40618-019-01090-x

10.1016/j.pcd.2019.06.007

10.3760/cma.j.issn.0253-9624.2018.12.016

10.1007/s15006-020-4352-5

10.1111/dme.14358

10.1111/jdi.13619

10.3389/fendo.2021.779636

10.1017/jns.2021.82
