# Supplementary material for: Meal frequency strategies for the management of type 2 diabetes subjects: A systematic review
Source: PLoS One. 2024 Feb 29;19(2):e0298531. doi: 10.1371/journal.pone.0298531 (PMC10903815; doi:10.1371/journal.pone.0298531)
Supplement: S4 File — (DOCX) [file pone.0298531.s004.docx]

**SEARCH TERMS**

(Meal frequency) AND (Diabetes Mellitus OR Type 2 Diabetes OR DMT2 OR T2D)

(Meal frequency) AND (Diabetes Mellitus OR Type 2 Diabetes OR DMT2 OR T2D) NOT (risk)

(Breakfast skipping) AND (Diabetes Mellitus OR Type 2 Diabetes OR DMT2 OR T2D)

(Snacks) AND (Diabetes Mellitus OR Type 2 Diabetes OR DMT2 OR T2D)

(Chrononutrition) AND (Diabetes Mellitus OR Type 2 Diabetes OR DMT2 OR T2D)

(Time-restricted Feeding) AND (Diabetes Mellitus OR Type 2 Diabetes OR DMT2 OR T2D)

From the last 10 years (2013-2023)

The search was limited to literature in English.
